# Biomaterials for bone tissue engineering scaffolds: a review

**DOI:** 10.1039/c9ra05214c

**Published:** 2019-08-21

**Authors:** Huawei Qu, Hongya Fu, Zhenyu Han, Yang Sun

**Affiliations:** School of Mechatronics Engineering, Harbin Institute of Technology Harbin 150001 China hongyafu@hit.edu.cn; School of Basic Medicine, Heilongjiang University of Chinese Medicine Harbin 150030 China sunyang@hljucm.net

## Abstract

Bone tissue engineering has been continuously developing since the concept of “tissue engineering” has been proposed. Biomaterials that are used as the basic material for the fabrication of scaffolds play a vital role in bone tissue engineering. This paper first introduces a strategy for literature search. Then, it describes the structure, mechanical properties and materials of natural bone and the strategies of bone tissue engineering. Particularly, it focuses on the current knowledge about biomaterials used in the fabrication of bone tissue engineering scaffolds, which includes the history, types, properties and applications of biomaterials. The effects of additives such as signaling molecules, stem cells, and functional materials on the performance of the scaffolds are also discussed.

## Abstract 2

Bone tissue engineering has been continuously developing since the concept of “tissue engineering” has been proposed. Biomaterials, as the basic material for the fabrication of scaffolds, play a vital role in bone tissue engineering.

## Graphical abstract

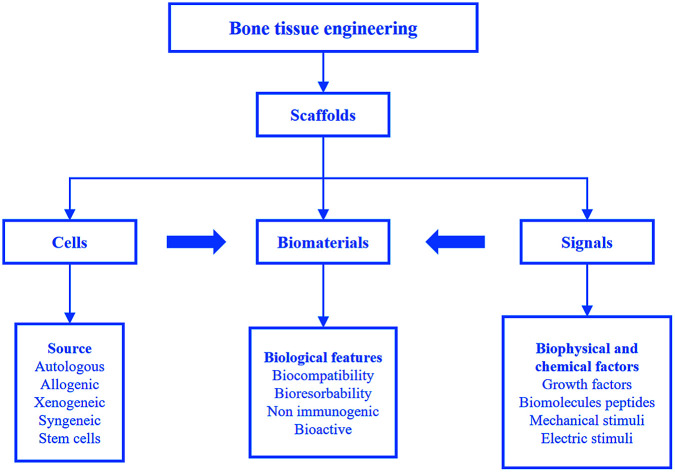

## 1. Introduction

Bone and its associated diseases, accounting for half of chronic diseases in people over 50 years old, still remain an important clinical challenge.^1,2^ Although bones have a certain healing and/or regeneration capacity, it cannot be accomplished by itself for large segmental bone defects. Large bone defects or injuries, caused by old age, traffic accident, fracture nonunion, bone tumor resection, *etc.*, are serious problems in orthopaedics, and they bring great harms to health and the quality of life.^3,4^ Autologous bone grafting is still regarded as the “gold standard” for repairing bone defects. However, the drawbacks of autologous bone grafting include secondary damages, high donor site morbidity, limitation of special shape, insufficiency of autogenous bone and so on. These weaknesses limit its widespread use in clinical settings.

The term “tissue engineering” was first used in 1987.^5^ It is the utilization of a combination of multidisciplinary approaches to improve or replace biological tissues. In recent years, with the rapid development of tissue engineering technology, bone tissue engineering has become a hopeful approach for repairing bone defects. Scaffolds play a crucial role in bone tissue engineering. Their purpose is to mimic the structure and function of the natural bone extracellular matrix (ECM), which can provide a three-dimensional (3D) environment to promote the adhesion, proliferation, and differentiation and to have adequate physical properties for bone repair. An ideal scaffold should be biodegradable, biocompatible, bioactive, osteoconductive and osteoinductive. Artificial bone scaffolds with biomaterials and additives, such as drugs, growth factors (GFs) and stem cells, have been useful for bone repair.

The biomaterials (biomedical materials), which are basic components of scaffolds, play an important role in bone tissue engineering. Archaeological findings showed that materials such as human or animal bones and teeth, corals, shells, wood, and several metals (gold, silver and amalgam) were used for the replacement of missing human bones and teeth.^6^ For example, in the ancient times, the Etruscans learnt to replace damaged teeth with artificial graft obtained from the bones of oxen. In the early 1960s, the limitations of biological bone substitute materials resulted in the emergence of a multidisciplinary field called “Biomaterials”.^7^ Biomaterials are used for the evaluation, treatment, augmentation, repair or replacement of tissues or organs of the body. Ancient alternative materials are mostly bioinert (biologically inert), and these materials interact less with the surrounding tissues and are even toxic to humans. An ideal biomaterial should be non-cytotoxic, printable, biodegradable, bioactive, and osteoconductive *in vivo*. Due to the various needs of scaffolds, composite materials composed of two or more materials with excellent properties are widely used in bone tissue engineering.

Numerous natural and synthetic polymers such as calcium phosphates, calcium carbonate, and bioactive glasses have been used to fabricate scaffolds. Recent outstanding approaches include the addition of conductive polymers (CPs), inducerons (signaling molecules, unlike bone morphogenetic protein 2 (BMP-2)) and mechanical signals (elastic polymer networks such as hydrogels) to bone tissue engineering scaffolds. With the integration, intercrossing and development of the fields of medicine, biology, materials and other disciplines, biomaterials have been extensively used in the fabrication of bone tissue engineering scaffolds.^8^

This article gives a brief introduction to the descriptions of the hierarchical structure, chemical composition of natural bone and strategies for bone tissue engineering. It aims to outline the history, types, properties and development methods of common biomaterials used to fabricate scaffolds. Further, the review also highlights the biomaterial scaffolds with additives. Finally, it examines the combination of advanced technology and biomaterials, and emphasizes the challenges and opportunities of biomaterials in bone tissue engineering scaffolds.

## 2. Materials and methods

All studies (*in vitro* and *in vivo*) concerning the application of biomaterials to manufacture scaffolds for bone tissue engineering were researched in duplicate in the Medline (PubMed) online database. The PubMed search was performed to look for articles published in English between January 1, 2010 and January 1, 2019. The Medical Subject Heading (abbreviated as MeSH) terms “bone and bones”, “biocompatible materials” and “tissue scaffolds” were used together with the keywords “bone tissue engineering”, “biomaterials” and “scaffolds” to apply the following search strategy:

(((“Bone and Bones[Mesh] OR (bone[All Fields]) OR (bones[All Fields]) OR (“bones and bone”[All Fields]) OR (“bones and bone tissues”[All Fields]) OR (bone[All Fields] AND (tissue[All Fields] OR tissues[All Fields])) OR (“bone tissue”[All Fields]) OR (“bone tissues”[All Fields])) AND (“biocompatible materials”[Mesh] OR (“biocompatible materials”[All Fields]) OR ((material[All Fields]) AND (biocompatible[All Fields])) OR (biomaterials[All Fields]) OR (biomaterial[All Fields]) OR (“bioartificial materials”[All Fields]) OR (“bioartificial material”[All Fields]) OR ((material[All Fields]) AND (bioartificial[All Fields]))) AND (“tissue scaffolds”[Mesh] OR ((scaffold[All Fields] OR scaffolds[All Fields] OR scaffolding[All Fields] OR scaffoldings[All Fields]) AND tissue[All Fields]) OR (“tissue scaffold”[All Fields]) OR (“tissue scaffolding”[All Fields]) OR (“tissue scaffoldings”[All Fields]))) OR ((bone tissue engineering) AND (biomaterials) AND (scaffolds))) AND (“2010/01/01”[Date-Publication]: “2019/01/01”[Date-Publication]).^9,10^

The follow-up period or sample size is not limited. Meta analyses and systematic reviews were not included. Scientific research regarding the following topics was not considered: scaffolds for assisted positioning of transplants and help with surgical planning before the surgery.

### 2.1 Study selection

Two of the authors individually selected the titles and abstracts of the articles obtained by the above-mentioned search. Then, the selected studies were independently carefully sifted by both of the reviewers. Any disagreement was determined through discussions between them.

### 2.2 Data extraction

Two of the authors separately summarized the search and sought consensus among other authors in the process. The undermentioned information was recorded: the publication information including the author's name and publication data, the biomaterials applied to manufacture scaffolds and their important characteristics.

## 3. Structure, mechanical properties and materials of natural bone

### 3.1 Hierarchical structure of bone

**Fig. 1:**
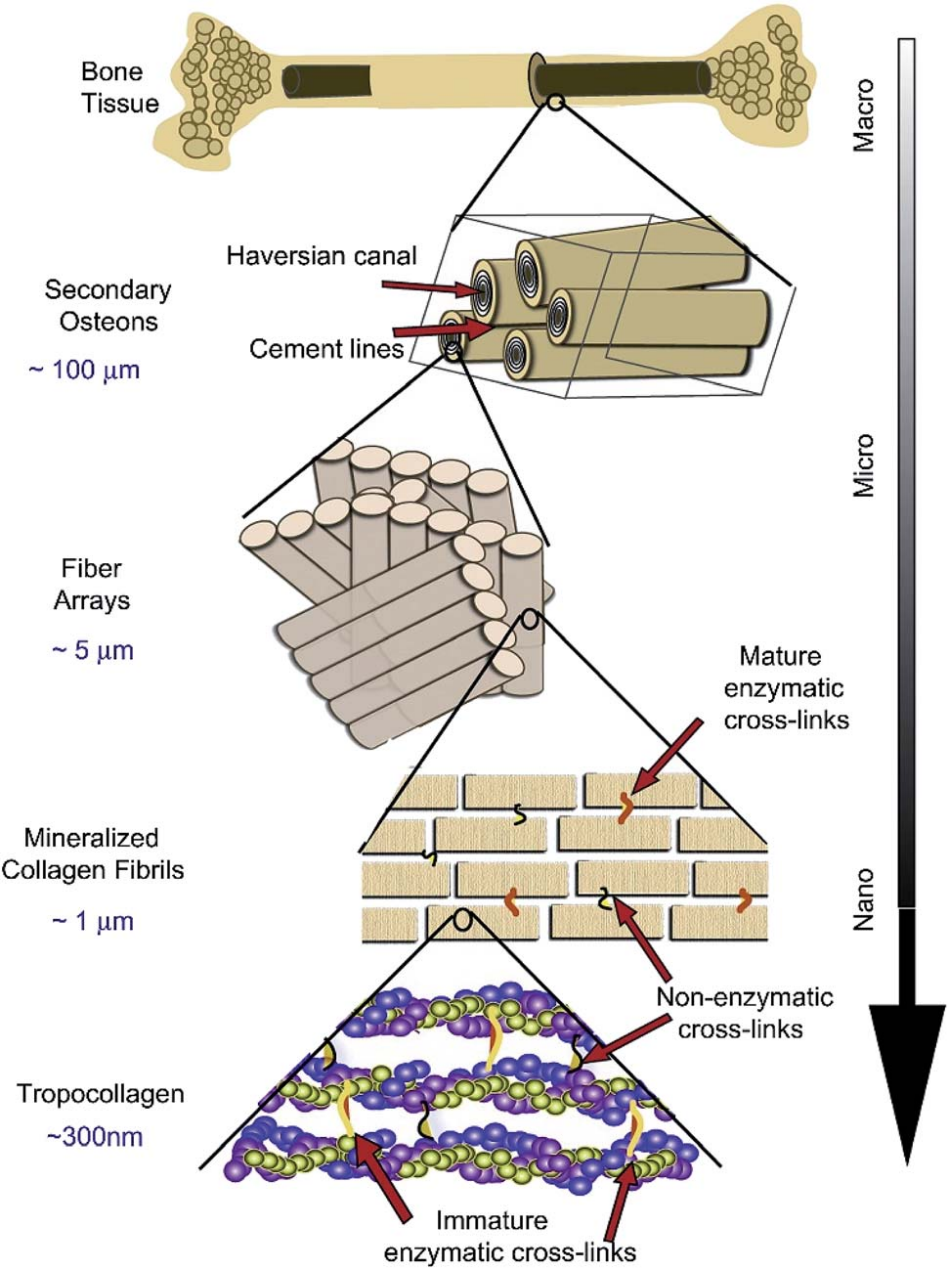
Hierarchical structure of natural bone. Reproduced from ref. 11 with permission from Elsevier, copyright 2011.

As the main part of the human skeletal system, bone plays a crucial role in providing structure, supporting mechanical movement, protecting organs, and producing and hosting blood cells. It has a complex hierarchical structure based on the length and width scale, which consists of the macro scale (trabecular bone, also known as cancellous or spongy bone, and compact bone, also named cortical bone), microscale and sub-microscale (haversian canals, osteons and lamellae), nanoscale (fibrillar collagen) and sub-nanoscale (such as minerals, collagen and so on), as shown in Fig. 1.^11^ The structure of natural bone has been presented in various articles.^11–21^ Compact bone is nearly solid, except for ∼3–5% of rooms for canaliculi, osteocytes and so on.^18^ However, trabecular bone is an interconnected porous network and has a higher bone surface-to-bone volume (BS/BV) ratio than compact bone.

### 3.2 Mechanical properties of bone

The mechanical properties of natural bone vary greatly with respect to age and the body part. Young's modulus and yield stress of natural bone are anisotropic. A complete understanding of the mechanics of living bones remains an important scientific challenge. Table 1 shows the mechanical properties of natural bone obtained from the reported data.^18^ The longitudinal direction of the compact bone is robuster and stiffer than its transverse direction. The trabecular bone has a porous structure, and the porosity and arrangement of the individual trabeculae determine its mechanical properties.

**Table 1:** Mechanical properties of natural bone

	Modulus (GPa)		Strength (MPa)		Poisson's ration
Compact bone	Longitudinal	17.9 ± 3.9	Tension	135 ± 15.6	0.4 ± 0.16
			Compression	205 ± 17.3	
	Transverse	10.1 ± 2.4	Tension	53 ± 10.7	0.62 ± 0.26
			Compression	131 ± 20.7	
	Shear	3.3 ± 0.4	Shear	65 ± 4.0	
Trabecular bone	Vertebra	0.067 ± 0.045		2.4 ± 1.6	
	Tibia	0.445 ± 0.257		5.3 ± 2.9	
	Femur	0.441 ± 0.271		6.8 ± 4.8	

### 3.3 Natural composition of bone

The understanding of the material components of natural bone plays a crucial role in the selection of scaffold materials. Natural bone consists of cells, ECM assembled from collagen fibrils and hydroxyapatite (HA), and bound minerals. Collagen and HA together account for ∼95% of natural bone under dry conditions.^21^ The composition of natural bone is presented in Table 2.^19^ Biological apatites deviate from the stoichiometric composition of HA and contain certain amounts of ion substitution impurities such as Na^+^, Mg^2+^, Cl^−^, K^+^, F^−^, and Zn^2+^. HA is the major inorganic component of human skeleton.

**Table 2:** Chemical composition of bone (wt%)

Inorganic Phase	Organic Phase
HA ≈ 60	Collagen≈20
H_2_O ≈ 9	Noncollagenous proteins≈3
Carbonate ≈ 4	Traces: polysaccharides, lipids, and cytokines
Citrate ≈ 0.9	Primary bone cell: osteoblasts, osteocytes, and osteoclasts
Na^+^ ≈ 0.7	
Mg^2+^ ≈ 0.5	
Cl^−^	
Others: K^+^, F^−^, Zn^2+^, Fe^2+^,Cu^2+^,Sr^2+^, and Pb^2+^	

## 4. Bone tissue engineering

**Fig. 2:**
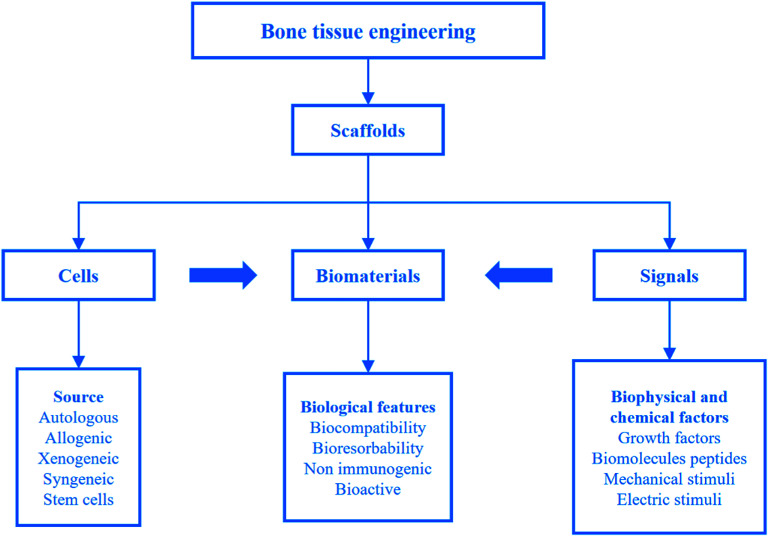
Strategies for bone tissue engineering. Reproduced from ref. 22 with permission from Springer, copyright 2018.

Although human bones have a certain self-healing ability, they are powerless for large bone defects. To overcome the problems, bone tissue engineering is proposed on the basis of tissue engineering. Bone tissue engineering aims to induce new tissue repairing and regeneration by the synergy of cells, signals and scaffolds.^8^ A scaffold composed of biomaterials is a carrier of cells and signals. It plays a key role in bone tissue engineering. Strategies for bone tissue engineering are shown in Fig. 2.^22^

For the large-sized tissues and origins with different shapes, it is necessary to design a temporary support to provide spaces for cell proliferation, differentiation and growth. The support is called scaffold, transplant, template or artificial ECM. As noticed before, an ideal scaffold should have biocompatibility, suitable mechanical properties, high porosity and gradient pore structure. As the new tissue grows, the implanted scaffold gradually degrades until the new tissue completely replaces it. The design and fabrication of scaffolds with customization can be obtained by computer-aided design and computer-aided manufacturing (CAD/CAM) technology. Biomaterials are an important part of the scaffolds, and an ideal biomaterial should possess the following characteristics: (1) biocompatibility; (2) biodegradability; (3) easy printing and processing. During the last decades, researchers have shown increasing interest towards biomaterials for their application in bone tissue engineering scaffolds.

Generally, the obtained scaffolds should be biologically investigated. The main approaches of biological research *in vitro* as forecasting test before pre-clinical can be divided into two main categories: (1) *in vitro* culture experiments such as scaffold toxicity tests, animal or human cells (such as BMSCs,^23^ hMSCs,^24^*etc.*) and (2) *in vivo* animal experiments (such as repairing of femur defects in rats).^25^ Scaffolds with non-toxic, good biocompatibility are the basis of bone repair and regeneration, in which biomaterials play an important role in the excellent performance of the scaffolds.

## 5. Various biomaterials for bone tissue engineering scaffolds

### 5.1 History of biomaterials

In the long history of human development, tissues and organs have evolved with respect to function after millions of years, but humans have been using artificial substitutes to repair damaged tissues only for decades. In the year 659 AD, the Chinese first used dental amalgam to repair defects in teeth.^26^ The limitations of bone replacement materials have resulted in the utilization of synthetic alternative materials for bone repair, replacement and enhancement. “Biomaterials” appeared in the early 1960s.^7^ The history of using biomaterials for scaffolds based on three different generations is briefly introduced below.^8^

The first generation of biomaterials appeared in the 1960s.^27^ It aimed to achieve the performance of the biomaterial to match the replaced tissue with the least toxic reaction to the host. They are generally bioinert, and interact minimally with the surrounding tissues. The first generation of biomaterials mainly includes: metals (such as titanium or titanium alloys), synthetic polymers (such as PMMA and PEEK) and ceramics (such as alumina and zirconia).

The most important feature of the second-generation biomaterials is their bioactive nature, and some could be biodegradable *in vivo*. They consist of synthetic and natural polymers (*e.g.* collagen), calcium phosphates, calcium carbonate, calcium sulfates, and bioactive glasses.

The third generation of biomaterials are designed to induce specific beneficial biological responses by the addition of instructive substances based on the second-generation biomaterials with excellent properties and/or new biomaterials with outstanding performance. Some of the instructive substances include, but are not limited to, biological factors or external stimuli.

### 5.2 Simple biomaterial scaffolds

**Fig. 3:**
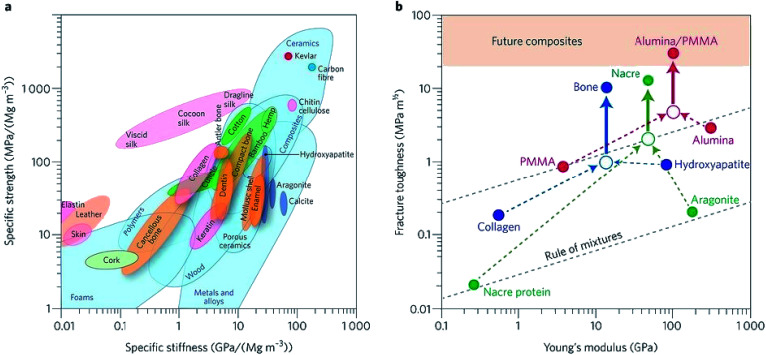
Performance of natural and synthetic materials. (a) Ashby chart of strength and stiffness for natural and synthetic materials. (b) Calculation for natural and synthetic materials. Reproduced from ref. 17 with permission from Nature Publishing Group, copyright 2014.

Biomaterials such as metals, natural polymers, synthetic polymers, ceramics, and their composites have been widely used in biomedical fields for decades. Fig. 3a indicates the values (normalized by density) of stiffness and the strength of various materials by an Ashby plot.^17^ Natural materials, except silk that exhibits excellent toughness, have much lower values of strength and toughness than engineering materials. However, many natural materials have a toughness value that far exceeds their composition and their homogeneous mixture (as shown by the dashed line in Fig. 3b).^17^ Selection of matrix material plays a crucial role in the properties of bone scaffolds. Various polymers have been developed to fabricate bone tissue engineering scaffolds. An overview of different biomaterials including their characteristics, advantages, and disadvantages is given in Table 3.

**Table 3:** Outlined characteristics of various biomaterials used to fabricate bone tissue engineering scaffolds

Biomaterials	Characteristics	Advantages	Disadvantages	Ref.
*Metal*	Suitable mechanical properties of biocompatible metallic scaffolds	Outstanding mechanical properties	Non-biodegradable	
		Biocompatible	Corrosion	
Tantalum	Bioactive and corrosion resistance	Extensively used as implant biomaterials	Almost no degradation lead to a second surgery for removing the implant	28–32
Magnesium	Good porous and biodegradable implant	Mechanical properties similar to human bone	Toxicity risk caused by metal ion or particle leaching	33–39
		Biodegradable		
Titanium and titanium alloys	Durable, biocompatible, highly corrosion resistant and very similar modulus of elasticity for trabecular bone	High bone affinity	Non-biodegradable	40–44
Nickel-titanium alloy (nitinol)	Particular mechanical properties (such as the shape memory and superelastic effects)	Low modulus of elasticity, pseudo-elasticity, and high damping capacity, better match the properties of natural bone better than any other metals	Almost no degradation for nitinol, the relatively high stiffness of titanium can cause stress shielding and implant loosening	45–48
*Natural polymer*	Similarity to ECM, specific degradation rates and good biological properties	Biocompatible	Low mechanical strength	
		Degradation		
Collagen	Important part of natural bone organic materials. Excellent biocompatibility	Biodegradable	Disinfection and handling are relatively difficult	49–51
		Various forms of scaffolds (*e.g.*, sheets)		
Gelatin	Denaturalized collagen	Forming blends through cross-linking		52–55
Silk fibroin	Silk fibroin with outstanding mechanical properties			56–58
Chitosan	Polysaccharide with positive charge, biocompatibility and resistance to bacteria			59
Alginate	Polysaccharide with negative charge, and can crosslink and print by injection			60–62
Hyaluronic acid	Glycosaminoglycan with negative charge, biocompatibility, forming hydrogel through cross-linking	Ease to chemical functionalization and degradability		58,63–66
*Synthetic polymer*		Changeable mechanical and physical properties	Possible adverse tissue reactions caused by acidic degradation	
PLA, PGA and PLGA	FDA-approved materials for clinical applications	Water solubility and crystallinity tunable by changing hydroxylation degree	Non-hydrophobic and shortage of cell adhesion	67–69
PCL	Excellent crystallinity and mechanical properties	An crosslink *in situ* and print by injection	Degradation rate in years	70–73
PVA	Hydroxylated synthetic polyvinyl acetate	Ability to manufacture implants with various characteristics such as shape, porosity and degradation rate		74–77
PPF	Has numerous nonsaturable double bonds and the crosslinks may be toxic	Adjustable mechanical strength and rates of degradation		78,79
Polyurethane (PU)	Remarkable mechanical properties			80–82
*Bioinert ceramic*	Cannot perform medical reactions with living tissue after implantation			
Aluminum, *e.g.*, α-aluminum oxide (Al_2_O_3_)	Improve mechanical properties; lack of biological activity			83–86
Zirconia	Interconnected structures; lack of chemical bonds and biological reactions between living tissues			87–89
*Bioactive ceramic*	Can show medical reactions with living tissue after implantation			
HA	The main inorganic component of natural bone	Highly biocompatible, non-toxic and osteoconductive		6,85,90,91
Tricalcium phosphate (TCP), *e.g.*, beta-tricalcium phosphate (β-TCP)	The ratio of calcium to phosphorus is close to natural bone tissue	Biocompatibility, no rejection and can provide calcium and phosphorus for new tissue	α-TCP has excessive dissolution and rapid degradation	56,92–95
			Degradation rate and osteogenic speed are inconsistent	
Calcium sulfate (CaSO_4_)	CaSO_4_ is a good material to choose after tumor resection			96–99
Akermanite (ca, Si, Mg)	Excellent mechanical properties and controllable degradation rate			100–102
	Better osteogenic differentiation and increased gene expression compared to β-TCP			
Diopside (MgCaSi_2_O_6_)	Low temperature and fast firing and good thermal expansion properties			103–106
Bioactive glasses (BGs)	The main components for Na_2_O, CaO, SiO_2_ and P_2_O_5_; brittleness			107–113

### 5.3 Composite biomaterial scaffolds

Composite biomaterials are designed to combine two or more materials. The purpose of using composite materials is mainly to improve the processability, printing performance, mechanical properties and bioactivity of the scaffolds. Ti6Al4V, HA, β-TCP and BG are widely used as bioactive biomaterials due to specific biological reactions between scaffolds and living tissues. Bioresorbable biomaterials applied in bone tissue engineering are generally natural polymers (such as collagen, gelatin, silk fibroin, and chitosan), synthetic polymers (such as PLA, PGA, and PCL) and ceramic (such as HA, β-TCP, and BGs). Scaffolds containing additives (such as GFs) have been used in clinical applications because of their excellent bone regeneration capabilities. The general composite biomaterial scaffolds with additives (signaling molecules, stem cells, functional materials, and so on) for bone tissue engineering are summarized in Table 4, which include metal matrix composites, polymer matrix composites, ceramic matrix composites, and functional composites.

**Table 4:** Summary of composites materials used to manufacture scaffolds for bone tissue engineering

Type	Raw materials	Additives	Study outcome	Ref.
Metal matrix composites	Ti6Al4V		Young's modulus similar to human natural bone, improved the mechanical shielding	114,115
	Ti6Al4V	Tantalum (Ta)	Better bone ingrowth in Ta-coated scaffolds	116
	Ti6Al4V	Simvastatin/Hydrogel	Significantly improved neovascularization, osteointegration and bone ingrowth	117
	Ti6Al4V	HA/pDA	Significantly promoted bone regeneration and improved osteointegration and osteogenesis	118
	Ti6Al4V/Fibrin glue	Vascular endothelial growth factor (VEGF) and BMP-2	Significantly enhanced both osteogenesis and angiogenesis for a single factor or dual factors, but synergistic effects of two-factor combination can observe angiogenesis but lack osteogenesis	119
Polymer matrix composites	Bioactive glass (BG)	Collagen-glycosaminoglycan (CG)	Promoting bone tissue regeneration and overcoming the problem of inadequate graft vascularization in tissue engineering	120
	Poly(L/DL lactide) (PLDL)/PCL	Osteogenon-drug	Use of osteogenon improves mineralization, cell adhesion and cell differentiation	121
	PEG/PU	BMSCs	The polymer matrix is highly thermally stable, regulatable, degradable at an acidic pH (5.8), biodegradable, cell compatible and has excellent porosity	23
	PLA	Bioactive organically modified glass (ormoglass)	The fibers are coated with different ormoglass components and their properties (roughness, stiffness and morphology) are adjusted by altering the trial parameters	122
	Poly(D,l-Lactide) (PDLLA)	BGs and CuO/ZnO	By appropriately adding Cu- and Zn-doped BG in the PDLLA, composite scaffolds can be obtained with improved bioactivity	123
Ceramic matrix composites	Titanium dioxide	PLGA/gentamicin	Confirmed the effective antibacterial activity of the released gentamicin and the compatibility of the scaffold on osteoblast-like cells(MG-63)	124
	HA/β-TCP	BMP-2	Real application possibilities for bone tissue engineering purposes	125
	β-TCP	Iron-containing	Iron maybe help to promote the bone conduction properties of calcium phosphate (CaP) ceramics	126
	n-HA/poly(D,L-lactide-co-glycolide) (PLAGA)	hMSCs	Allowing for the generation of engineered bone tissue	24
	HA/Poly(D,l-lactic acid)-co-poly-(ethylene glycol)-co-poly(D,L-lactic acid) (PELA)	BMSCs/rhBMP-2	Making the scaffolds suitable for evaluating bone regeneration approaches based on cell/the PELA/HA scaffolds with 500 ng of rhBMP-2	25
Functional composites	Photocrosslinking of PCL and bioactive polydopamine coating	Temperature	The capacity to automatically fit into irregular defects and superior bioactivity because of polydopamine-coating	127
	Polypyrrole (PPy), HA, gelatin and mesoporous silica	Electrical stimulation	Good mechanical properties, higher protein adsorption	128
	PLGA	Black phosphorus (BP)/SrCl_2_	The obtained scaffolds had good biocompatibility and good bone regeneration ability under near-infrared (NIR) irradiation *in vivo* in rats	129
	Polypyrrole/alginate (PPy/Alg)	hMSCs and electrical stimulation	Enhanced cell adhesion and growth	130
	Gelatin/bioactive glass	Poly(3,4-ethylenedioxythiophene):poly(4-styrene sulfonate) (PEDOT:PSS) and electrical stimulation	Adding PEDOT stabilizes the structure of scaffolds and enhances the cellular properties of mesenchymal stem cells	131
	Transglutaminase cross-linked gelatin (TG-Gel)	BMP-2, matrix rigidity and mechanical signaling	The combination of hydrogel hardness and BMP-2 has a synergistic effect on cellular osteogenic differentiation	132

Bioactive metal matrix composites are widely used in clinical medical settings because of their outstanding mechanical properties, excellent biocompatibility, thermal stability, and corrosion resistance. Titanium, tantalum and their respective alloys are considered to be the preferred biomaterials for scaffolds. However, the high costs of manufacturing scaffolds limit their widespread development. Ti6Al4V is an outstanding representative of metal matrix composites. Young's modulus of the suitable porous Ti6Al4V scaffolds can be similar to natural bone and improve the mechanical shielding to the living tissue.^114,115^ The Ti6Al4V scaffolds can significantly increase bone ingrowth, osteointegration, and osteogenesis by covering the tantalum coating,^116^ adding simvastatin/hydrogel,^117^ or polydopamine-assisted hydroxyapatite coating (HA/pDA),^118^ as summarized in Table 4. Although metal matrix composites, such as Ti6Al4V, have many outstanding advantages; the non-biodegradable properties of metal matrix composites fundamentally limit their potential to become ideal materials.

**Fig. 4:**
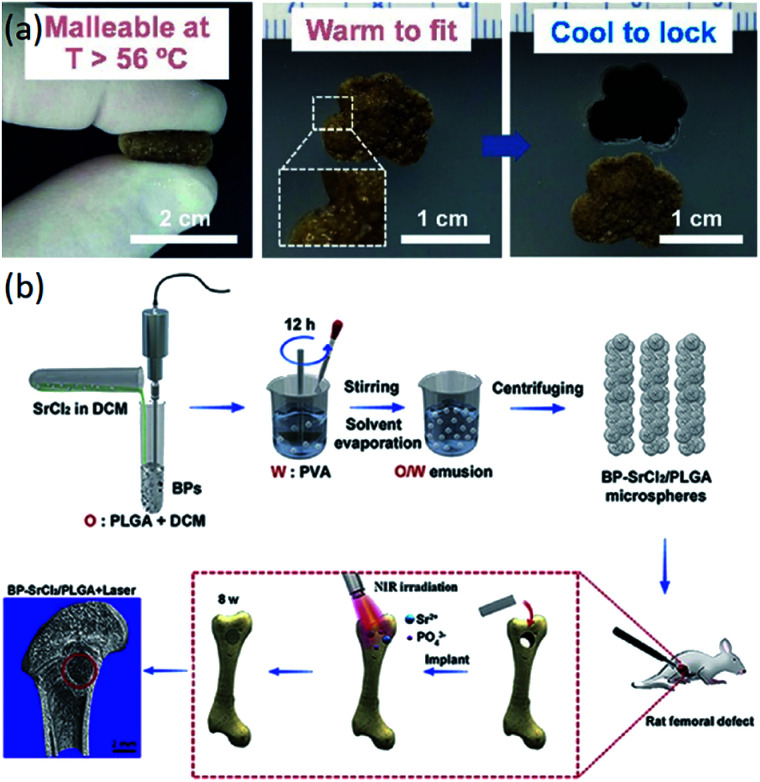
Functional composite bone tissue engineering scaffolds. (a) Effect of temperature on the scaffolds. Reproduced from ref. 127 with permission from Elsevier, copyright 2014. (b) Effect of near-infrared light on the scaffolds. Reproduced from ref. 129 with permission from Elsevier, copyright 2018.

In recent years, the application of polymer matrix composites and ceramic matrix composites has made great progress in bone tissue engineering scaffolds. Polymer composites have various excellent properties, such as biodegradability and mechanical properties.^122–131^ Ceramic materials, especially HA, are the main inorganic constituents of natural bone.^19^ Composite materials composed of ceramic materials and polymer materials have desirable properties for the manufacturing of scaffolds for bone tissue engineering.^125,126,130,131^ The composite scaffolds with additives (signaling molecules, stem cells and functional materials) have superior performance compared to just composite scaffolds (Table 4). The composite scaffolds with additives could further enhance the performance of the scaffolds. As shown in Fig. 4a, scaffolds with bioactive polydopamine coating have the capacity to automatically fit into irregular defects at higher temperatures. Wang *et al.* fabricated BP-SrCl_2_/PLGA scaffolds for rat femoral defects, and the near-infrared light-triggered platform significantly enhanced bone regeneration, as seen in Fig. 4b.^129^

## 6. Conclusion

In this paper, the summarized literature, which involves biomaterials for bone tissue engineering scaffolds, has been reviewed. The application and properties of various biomaterials used to fabricate scaffolds have also been elaborated. In particular, composite materials such as metal matrix composites, polymer matrix composites, ceramic matrix composites, and functional composites have been discussed. It was found that additives such as signaling molecules, stem cells, and functional materials can enhance the performance of the scaffolds. Although it was impossible forty years ago to find a material that is not repelled by living tissue, nowadays biomaterials have been used for bone repair. Improved performance of ideal biomaterials is required for their positive interactions with host tissues. The approaches for bone regeneration will make giant steps with the exploitation of novel biomaterials and new strategies, particularly the deep integration of nanotechnology, stem cell science and other fields.

## Abbreviations

3D: Three-dimensional
ECM: Bone extracellular matrix
GFs: Growth factors
CPs: Conducting polymers
MeSH: Medical subject heading
BS/BV: Bone surface to bone volume
HA: Hydroxyapatite
n-HA: Nano-hydroxyapatite
CAD: Computer-aided design
CAM: Computer-aided manufacturing
PMMA: Poly(methyl methacrylate)
PEEK: Polyether ether ketone
PLA: Poly(lactic acid)
PGA: Poly(glycolic acid)
PLGA: Poly(lactic-*co*-glycolic acid)
PVA: Poly(vinyl alcohol)
PPF: Poly(propylene fumarate)
PU: Polyurethane
Al_2_O_3_: α-Aluminum oxide
TCP: Tricalcium phosphate
β-TCP: beta-tricalcium phosphate
CaP: Calcium phosphate
CaSO_4_: Calcium sulphate
HA/pDA: polydopamine-assisted hydroxyapatite coating
Ta: Tantalum
CG: Collagen-glycosaminoglycan
PLDL: Poly(L/DL lactide)
BP: Black phosphorus
NIR: Near-infrared
BMSCs: Bone marrow stromal cells
PEG: Poly-(ethylene glycol)
PDLLA: Poly(d,l-Lactide)
BMP-2: Bone morphogenetic protein 2
VEGF: Vascular endothelial growth factor
PELA: Poly(d,l-lactic acid)-*co*-poly-(ethylene glycol)-*co*-poly(d,l-lactic acid)
PLAGA: Poly(d,l-lactide-*co*-glycolide)
rhBMP-2: Recombinant human bone morphogenetic protein-2
PPy/Alg: Polypyrrole/alginate
hMSCs: Human mesenchymal stem cells
PEDOT: PSS poly(3,4-ethylenedioxythiophene): poly(4-styrene sulfonate)
TG-Gel: Transglutaminase cross-linked gelatin

## Conflicts of interest

There are no conflicts to declare.

## Supplementary Material
